# Multi-Enzyme Supplementation Modifies the Gut Microbiome and Metabolome in Breeding Hens

**DOI:** 10.3389/fmicb.2021.711905

**Published:** 2021-12-03

**Authors:** Yuchen Liu, Dan Zeng, Lujiang Qu, Zhong Wang, Zhonghua Ning

**Affiliations:** ^1^National Engineering Laboratory for Animal Breeding and Key Laboratory of Animal Genetics, Breeding and Reproduction, Ministry of Agriculture and Rural Affairs, College of Animal Science and Technology, China Agricultural University, Beijing, China; ^2^Huayu Agricultural Science and Technology Co., Ltd., Handan, China; ^3^State Key Laboratory of Animal Nutrition, College of Animal Science and Technology, China Agricultural University, Beijing, China

**Keywords:** multi-enzyme, aged layers, immunity, reproduction performance, microbiome, metabolome

## Abstract

Laying and reproductive performance, egg quality, and disease resistance of hens decrease during the late laying period. Exogenous enzymes promote nutrient digestibility and utilization and improve the intestinal environment. However, the specific regulation of the gut microbiome and metabolome by exogenous enzymes remains unelucidated. This study was conducted to evaluate effects of dietary multi-enzyme supplementation on egg and reproductive performance, egg quality, ileum microbiome, and metabolome of breeders. Here, 224 Hy-Line Brown breeding hens (55 weeks old) were randomly allocated to two groups: dietary controls fed basal diet (DC), and test hens fed 0.2 g/kg corn enzyme diet (CE). Serum levels of total protein, globulin, immunoglobulin Y, and antibodies against the Newcastle disease virus and avian influenza H9 strain were significantly increased (*p* < 0.05). Egg albumen height, Haugh unit, and fertilization and hatching rates were also significantly increased (*p* < 0.05) in the CE-fed group. 16S rRNA sequence analysis showed that CE strongly affected both α- and β-diversity of the ileal microbiota. LEfSe analysis revealed that the potentially beneficial genera *Lactobacillus*, *Enterococcus*, *Faecalicoccus*, and *Streptococcus* were enriched as biomarkers in the CE-fed group. Microbial functional analysis revealed that the functional genes associated with harmful-substance biodegradation was significantly increased in the CE-fed group. Additionally, Spearman correlation analysis indicated that changes in microbial genera were correlated with differential metabolites. In summary, dietary multi-enzyme addition can improve egg quality, humoral immunity, and reproductive performance and regulate the intestinal microbiome and metabolome in breeders. Therefore, multi-enzymes could be used as feed additive to extend breeder service life.

## Introduction

With increasing age, physiological function and digestive enzyme activity decrease and are always accompanied by gut microbiota disorder after the peak laying period in breeding hens, causing significant economic loss (Liu et al., 2013; Jing et al., 2014; Gu et al., 2021). Exogenous addition of enzymes was considered to improve the degradation of harmful macromolecules and activity of endogenous enzymes to assist in the degradation of starch and protein (Gu et al., 2021).

Starch is a complex polysaccharide composed of amylose and amylopectin (AP). AP accounts for 70–80% of most starch sources and requires pullulanase for hydrolysis (Scott et al., 2013; Yin et al., 2018). Pullulanase is an important debranching enzyme that originates from bacteria, plants, and less commonly, fungi. Specifically, it could often attack α-1,6 linkages, thereby efficiently converting branched polysaccharides into small molecular sugars (Hii et al., 2012; Tomasik and Horton, 2012). In contrast to pullulanase, α-amylases split the α-1,4 glycosidic linkages in amylose to yield maltose and glucose (Sarian et al., 2017). Studies have demonstrated that the addition of α-amylase to a corn-soybean diet can release more feed energy and significantly improve apparent nutrient digestibility, digestive enzyme activity, and production performance of poultry (Aderibigbe et al., 2020). Glucoamylase (also known as amyloglucosidase or AMG) is an important digestive enzyme that mainly saccharifies partially processed starch/dextrin to glucose, which helps poultry absorb nutrients (da Costa Luchiari et al., 2021). Previous research indicated that supplementation with glucoamylase or protease combined with amylase could improve starch digestibility and gut microbiota diversity and promote the growth of broilers (Yin et al., 2018). Proteases can enhance protein and amino acid digestibility and reduce the adverse effects of heat-stabilized trypsin inhibitors or lectins, thus improving forage quality (Cowieson et al., 2017; Walk et al., 2018). A significant increase in the ileal digestibility of protein and amino acids occurs with proteases in poultry diets (Romero et al., 2014). Overall, exogenous enzymes can communicate with the host by utilizing indigestible dietary components and providing nutrients to regulate digestive, immune, and antioxidant functions to facilitate production performance and benefit the host (Pan and Yu, 2014; Cowieson and Kluenter, 2019; Monier, 2020; Giacobbo et al., 2021).

The use of enzymes in poultry feed is not uncommon. However, the role of enzymes in feed digestibility, productivity, and health of chickens is influenced by several factors, including the source, type, characteristics, dosage, and composition of complex enzymes as well as the diet structure, composition, and physiological status of chickens. In this study, we first evaluated the effects of new multi-enzymes (proteases, pullulanase, α-amylase, and glucoamylases) on laying performance, egg quality, reproductive performance, and immunity of older breeding hens and investigated the underlying mechanism through in-depth microbiome and metabolome analyses. Our objective was to develop a new nutritional strategy to improve health and extend the service life of breeding hens in their later laying stage.

## Materials and Methods

### Birds, Diets, and Management

The Animal Welfare Policy has approved the bird management and handling procedures. All animal procedures were performed according to the principles of the Animal Care and Use Committee of the China Agricultural University. A total of 224 Hy-Line Brown breeding hens (55-week-old) with similar production performances and weights were randomly divided into two treatment groups with seven replicates of 16 hens each (4 hens per cage, 40 cm wide, 62 cm long, and 45 cm high). One is the dietary control fed with basal diet (DC), and the other with 0.2 g/kg corn enzyme diet (CE). The CE diet contained 11,000 u/g proteases, 20 u/g pullulanase, 1,000 u/g α-amylase, and 1,000 u/g glucoamylases, and was provided by the Wuhan SunHY Biological Co., Ltd. All hens were handled following the Hy-Line Brown Laying Hens Management Guide, and the hens were housed at the HuaYu Poultry Breeding Co., Ltd. (Handan, Hebei). All experimental hens were vaccinated with inactivated Newcastle virus (NDV) plus avian influenza virus (H9 subtype) strain vaccine by intermuscular injection at 55 weeks of age. The essential diet is shown in Table 1 and meets the Chinese standards of agricultural trade standardization (NY/T33-2004).

**TABLE 1:** Ingredients and nutrient composition of basal diet.

Ingredients	Percent	Nutrient level^c^	Percent
Corn (CP 8.3%)	64.00	ME (MJ/Kg)	16.01
Soybean meal (CP 44.0%)	20.93	CP (%)	16.04
Soybean oil	0.70	CF (%)	3.24
Wheat bran	3.00	Methionine (%)	0.24
Limestone	9.50	Lysine (%)	0.70
Calcium hydrogen phosphate	1.00	Calcium (%)	3.49
Sodium chloride	0.30	Total P (%)	0.32
DL-Methionine (98%)	0.10		
L-Lysine HCL (78%)	0.07		
Vitamin premix^a^	0.03		
Mineral premix^b^	0.20		
Choline chloride (50%)	0.15		
Phytase	0.02		
Total	100.00		

*^*a*^Supplied per kilogram of complete diet: vitamin A, 13,500 IU; vitamin D3, 4,500 IU; vitamin E, 75 IU; vitamin K3, 3.6 mg; vitamin B1, 3.0 mg; vitamin B2, 9.24 mg; vitamin B6, 6.0 mg; nicotinic acid, 66 mg; pantothenic acid, 16.8 mg; biotin, 0.54 mg; folic acid, 2.10 mg; vitamin B12, 0.03 mg; vitamin C, 135 mg; choline, 675 mg; ethoxyquinoline, 15 mg.*

*^*b*^Mineral premix provided per kilogram of complete diet: iron, 80 mg; copper, 10 mg; manganese, 100 mg; zinc, 100 mg; iodine, 0.35 mg; selenium, 0.30 mg.*

*^*c*^CP and CF were measured values, and the other nutrients were calculated values.*

### Laying Performance Parameters

Eggs were collected daily during the experiment. The number of eggs laid, abnormal eggs, broken eggs, and egg weights were recorded on a replicate basis. The feed intake for each repetition was counted every 2 weeks. The average egg production rate, average egg weight, broken egg rate, abnormal egg rate, and feed egg ratio were calculated for 1–4, 5–8, and 1–8 weeks. Mortality was recorded daily as it appeared.

### Egg Quality Parameters

Ten eggs were randomly collected from each replicate (70 eggs/group) for internal and external quality analyses during the last 2 days of the experiment. An egg-shaped index tester was used to measure the egg length and shortest diameter. An eggshell color tester was used to measure the eggshell color value (Konicaminolta CM-2600d). A quasi-static compression device (Robotmation, Japan) was used to measure the eggshell breaking strength. After removing the inner shell membrane, the eggshell thickness was measured using a micrometer screw gauge at three different locations (lower, middle, and upper ends). Egg weight, albumen height, Haugh units, and yolk color were measured using an automatic egg quality analysis device (EMT-5200, Japan).

### Blood Biochemical Parameters

Blood samples were collected for analyzing blood biochemistry and detecting serum antibody titers for 1 day before the end of the experiment. After fasting for 8 h, one hen per replicate was randomly selected (a total of 7 hens/group), and whole blood was collected from the wing vein using sterile blood collection tubes. The blood was centrifuged at 3,000 rpm for 10 min. The serum was extracted into a sterile 2 mL centrifuge tube and stored at −20°C until detection. Serum was used to detect aspartate aminotransferase (AST), total protein (TP), albumin (ALB), globulin (GLB), albumin/globulin, high-density lipoprotein cholesterol (HDL-C), immunoglobulin Y (IgY), and total antioxidant capacity (T-AOC). All indexes were tested using kits purchased from the Nanjing Jiancheng Bioengineering Institute (Nanjing, China). Other serum samples were used to detect antibody titers of NDV and avian influenza H9 strains by hemagglutination and hemagglutination inhibition assays. The virus, antigen, and positive control sera were purchased from Qingdao Yebio Biological Engineering Co., Ltd.

### Reproductive Performance

All hens were inseminated on days 49 and 50 for 2 consecutive days of the formal phase. The semen was mixed and came from the same 12 cocks to ensure consistent semen quality. Eggs were collected on the 53rd–54th days. The total number of eggs produced and eligible hatching eggs were recorded and placed into pre-fumigated incubators. On the 18th day of incubation, the number of fertilized eggs was recorded by candling, and the eggs of identical replicates were placed in one string bag. On the 21st day of incubation, the number of newborn chicks in each replicate was recorded. Lastly, the rates of fertilized eggs and hatch of fertile (HoF) were calculated.

### Gut Microbiota Sequencing

One hen per replicate was randomly selected (a total of 6 hens/group, one sample less than the number of replicates was due to unqualified DNA amplification), and euthanasia was performed using carbon dioxide on the last day of this trial (56 days). The ileum contents from each bird were collected and immediately frozen in liquid nitrogen until DNA extraction. Microbial genomic DNA extraction was conducted according to the manufacturer’s instructions using the QIAamp 96 Powerfecal Qiacube HT Kit (5) (CatNo. 51531). DNA purity and concentration were detected using a NanoDrop 2000 spectrophotometer (Thermo Fisher Scientific, Waltham, MA, United States) and agarose gel electrophoresis. The purified DNA targeted the V3–V4 region of the 16S rDNA gene according to PCR bar-coded primers (343F: 5′-TACGGRAGGCAGCAG-3′ and 798R: 5′-AGGGTATCTAATCCT-3′). PCR was conducted using the KAPA HiFi Hot Start Ready Mix (KAPA Biosystems, Wilmington, MA, United States). Both reverse primers included a barcode and an Illumina sequencing adapter. The PCR products were visualized using 1% agarose gel electrophoresis, purified, and quantified using Agencourt AMPure XP beads (Beckman Coulter Co., United States) and Qubit dsDNA HS assay kit (Thermo Fisher Scientific), respectively. Sequencing was performed using an Illumina MiSeq platform with two paired-end read cycles of 300 bases each (Illumina Inc., San Diego, CA; OE Biotech Company, Shanghai, China).

### Bioinformatic Analysis of the Microbiome

Microbiota data were subjected to bioinformatics analysis using QIIME software (version 1.8.0) (Caporaso et al., 2010). Data quality filtering, ambiguous bases, low-quality sequence removal, paired-end read assembly, and detachment of chimeric sequences were conducted using QIIME (Caporaso et al., 2010), Trimmomatic (Bolger et al., 2014), FLASH (Reyon et al., 2012), and UCHIME algorithms (Edgar et al., 2011), respectively. Reads with a similarity threshold of ≥ 97% were assigned to the same operational taxonomic unit (OTU) using the Vsearch pipeline (Rognes et al., 2016). Taxonomy was assigned to the OTUs using the SILVA database (v.123) with the RDP classifier at a 70% confidence threshold (Quast et al., 2012). Alpha diversity (Chao1, Observed, Shannon, Simpson’s diversity) and beta diversity (principal coordinate analysis; PCoA) were calculated using QIIME 1.8 scripts.

Linear discriminant analysis (LDA) effect size (LEfSe) (Segata et al., 2011)^1^ was used to identify representative species. LDA was performed from the phylum to genus level, and LDA scores ≥ 4.0 and *p*-values < 0.05 were considered signature taxa and selected for plotting and further analysis. The predicted metagenomic functional content was determined using PICRUSt^2^ software by combining 16s rRNA data against the Greengenes database and the normalized data were analyzed to predict metagenomes using the Kyoto Encyclopedia of Genes and Genomes (KEGG) Orthology database.^3^ Pairwise statistical comparative analysis (Welch’s *t*-test, storey FDR correction) of microbial function was performed using STAMP (V2.1.3) (Parks et al., 2014). The microbial co-occurrence network analysis was performed using the CCLasso, sparCC, and NAMAP with Spearman correlation inference algorithm to elucidate gut microbiota interactions by MetagenoNets with default parameters (Nagpal et al., 2020). Only significant correlations (*p* < 0.05) based on the bootstrapping of 100 iterations were plotted.

### Untargeted Metabolomics by Liquid Chromatography-Mass Spectrometry

The ileal chyme (30 mg) was precisely weighed and transferred to 1.5 mL microcentrifuge tubes (Eppendorf), to which two 3 mm stainless steel beads were added. Then, 20 μL of L-2-chlorophenylalanine (0.3 mg/mL) and 17:0 Lyso PC (1-heptadecanoyl-sn-glycero-3-phosphocholine, 0.01 mg/mL) were used as the internal standard. Both were configured with methanol. An internal standard mixed with 400 μL of methanol aqueous solution (CH_3_OH: H_2_O, V: V = 4:1) was added to each sample and pre-cooled at −20°C for 2 min. The sample was then ground in a fully automatic sample fast grinding machine (60 Hz, 2 min; Shanghai Jingxin Industrial Development Co., Ltd., Shanghai, China) and placed in an ultrasonic bath with ice water for 10 min. The sample was placed in a −20°C refrigerator for 20 min before centrifugation at 13,000 rpm at 4°C for 10 min. The supernatant was removed with a syringe and filtered by passing through a 0.22 μm-membrane filter to an LC-MS vial and stored at −80°C for subsequent analysis by *LC-MS*. Water, acetonitrile, formic acid, and methanol were purchased from CNW Technologies GmbH (Düsseldorf, Germany). L-2-chlorophenylalanine was purchased from Shanghai Hengchuang Bio-Technology Co., Ltd. (Shanghai, China). LysoPC17:0 was purchased from Avanti (Avanti Polar Lipids Inc., United States). All solvents and chemicals were of analytical or high-performance LC grade.

Metabolomics analysis was conducted using the Dionex U3000 UHPLC system (Waltham, MA, United States) coupled to a high-resolution QE plus mass spectrometer (Thermo Fisher Scientific) to analyze the metabolic profiles of the positive and negative ion modes. The LC system was fitted with an ACQUITY UPLC BEH C18 (100 × 2.1 mm, 1.7 μm) and a binary gradient elution system consisting of A) water (containing 0.1% formic acid) and B) acetonitrile (containing 0.1% formic acid) by the following separation gradient: 0 min 5% B, 1 min 5% B, 11 min 100% B, 13 min 100% B, 13.1 min 5% B, and 15 min 5% B. The column temperature was 50°C, and the flow rate was 0.35 mL/min. The injection volume was 5 μL, and the samples were randomized to avoid systematic errors. The mass spectrometer conditions and parameters were as follows: spray voltage, 3,800 V in positive mode, and 3,000 V in negative mode; capillary temperature, 320°C; aux gas heater temperature, 350°C; sheath gas flow rate, 35 arbitrary units; Aux gas flow rate, 8 arbitrary units; mass range: 70–1,000 m/z; full ms resolution, 70,000; MS/MS resolution, 17,500; and NCE, 20 and 40.

*LC-MS* raw data were collected by UNIFI (version 1.8.1) and then processed using Progenesis QI (version 2.3) with the following threshold parameters: precursor tolerance of 5 ppm, product tolerance of 10 ppm, and production threshold of 5%. Metabolites were identified by retention time, exact mass, and tandem MS data against the Human Metabolome Project,^4^ Lipidmaps (v2.3)^5^ and METLIN^6^ databases. All metabolites with a percentage of missing values>50% and quality scores<30 were discarded by qualitative screening.

### Metabolome Bioinformatics Analysis

Metabolome data were subjected to bioinformatics analysis using the SIMCA software (version 14.0, Umetrics, Umeå, Sweden). Principal component analysis (PCA) and orthogonal partial least squares discriminant analysis (OPLS-DA) models and plots were constructed using SIMCA. Volcano plots were plotted using the R package ggplot2. The differential metabolites were converted from names to KEGG compound IDs using MetaboAnalyst software (version 5.0),^7^ CTS (Wohlgemuth et al., 2010), and MBRole software (version 2.0).^8^ These IDs were used as input files for metabolite set enrichment analysis using MetaboAnalyst 5.0 software [annotations: KEGG pathway; Organism: *Homo sapiens* (human)] and MBRole 2.0 software [annotations: KEGG pathway; Organism: *Gallus gallus* (chicken)]. We also applied the pathway topology analysis [annotations: KEGG pathway; Organism: *G. gallus* (chicken)] to verify our findings using MetaboAnalyst with the default setting. Considering the relative lack of lipid information in the KEGG database, the differential metabolites that were annotated in the LipidMaps database were enriched by LIPEA^9^ [annotations: KEGG pathway; Organism: *G. gallus* (chicken)]. Spearman’s correlation between the differential microbial biomarkers and metabolites and the three identified metabolites and six microbial biomarkers were analyzed using R software. Only correlation coefficients with an absolute value of | r| > 0.6 (Adj *P*-value < 0.05) were considered a significant relationship. Network visualizations were performed using Gephi software (version 0.9.2, The Gephi Consortium, Paris, France) (Dalcin and Jackson, 2018).

### Statistical Analysis

All graphs and data calculations were generated using R software (version 4.0.2), Prism8 (GraphPad, United States) software, and SPSS 24.0 (SPSS Inc., Chicago, IL, United States) software. Measurement data are expressed as the mean and standard error. A normal distribution and homogeneity of variance were performed. Comparisons between the two groups were performed using Student’s *t*-test when it conformed to normal distribution and homogeneity of variance; otherwise, the non-parametric Wilcoxon rank-sum test was performed. *P* < 0.05 were considered as significant and 0.05 < *p* < 0.1 was considered a trend.

## Results

### Production Performance and Egg Quality

The laying performance of breeding hens fed the CE diet is presented in Table 2. Egg production, egg weight ratio, damaged egg ratio, abnormal egg ratio, FCR, mortality, and feed intake were not affected by CE administration at 55–59, 59–63, and 55–63 weeks (*p* > 0.05). The egg quality results are presented in Table 3. CE administration significantly increased the egg albumen height and Haugh unit (*p* < 0.05) but weakened the yolk color (*p* < 0.05) compared with those in the DC-fed group at week 63.

**TABLE 2:** Effect of supplemental multi-enzyme on the performance of aged breeding hens.

Item	Egg production (%)	Egg weight (g)	Damaged egg (%)	Abnormal egg (%)	FCR^*a*^ (g feed/g egg)	Mortality (%)	Feed intake (g/d/hen)
**55–59 weeks**							
DC^b^	74.1	62.2	4.9	2.8	2.3	1.8	107.2
CE^c^	76.0	63.1	4.5	3.2	2.2	0.9	105.0
SEM	1.7	0.4	0.5	0.4	0.1	0.7	1.0
*P*-value	0.59	0.25	0.70	0.61	0.20	0.55	0.32
**59–63 weeks**							
DC^b^	76.5	62.6	4.4	5.2	2.4	0.9	115.3
CE^c^	77.2	63.8	4.7	4.3	2.4	3.6	115.4
SEM	1.8	0.3	0.7	0.4	0.1	0.8	1.2
*P*-value	0.86	0.08	0.83	0.33	0.52	0.11	0.95
**55–63 weeks**							
DC^b^	75.3	62.4	4.6	4.0	2.4	2.7	111.2
CE^c^	76.6	63.4	4.6	3.8	2.3	4.5	110.1
SEM	1.5	0.3	0.6	0.4	0.0	0.9	1.0
*P*-value	0.67	0.13	0.99	0.71	0.21	0.32	0.61

*^*a*^FCR, feed conversion ratio.*

*^*b*^DC, dietary control (basal diet).*

*^*c*^CE, basal diet + 0.2 g/kg complex enzymes.*

**TABLE 3:** Effect of supplemental multi-enzyme on the egg quality of aged breeding hens (*n* = 70/group).

Item	DC^1^	CE^2^	SEM	*P*-value
Egg index	1.3	1.3	0.0	0.36
L	59.2	59.2	0.3	0.93
a	18.7	18.5	0.2	0.53
b	30.1	29.6	0.1	0.07
Shell strength (kg/cm^2^)	4.0	4.0	0.1	0.67
Egg weight (g)	61.1	61.7	0.4	0.48
Yolk color	7.7^a^	7.0^b^	0.1	0.00
Egg albumen height	5.9^b^	6.2^a^	0.1	0.02
Haugh Unit	75.0^b^	77.5^a^	0.6	0.03
Eggshell thickness	0.4	0.4	0.0	0.67

*^*a,b*^Different superscript within a row means significantly different (P < 0.05).*

*^1^DC, dietary control (basal diet).*

*^2^CE, basal diet + 0.2 g/kg complex enzymes.*

### Blood Biochemical Parameters

**FIGURE 1:**
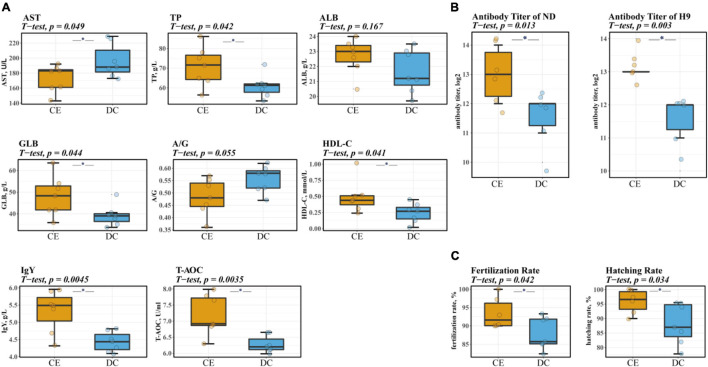
CE can increase the level of serum immunity, antioxidant, and liver-related indicators and significantly improve breeding hens’ reproduction performance (*n* = 7 hens/group). **(A)** The levels of biochemical parameters of breeding hens. **(B)** Serum antibody titers of NDV and avian influenza H9 strain of breeding hens. **(C)** The reproduction performance efficiency of breeding hens. Left: representative fertilized eggs rate; Right: representative the hatching of fertile (HoF) rate. ALB, Albumin; AST, Aspartate Aminotransferase; GLB, Globulin; HDL-C, High-density lipoprotein cholesterol; IgY, Immunoglobulin Y; ND, Newcastle disease. T-AOC, Total Antioxidant Capacity; TP, Total protein. *P < 0.05 compared to two groups.

Serum biochemical and antibody levels are physiological indices commonly used to evaluate animal health and immunity. CE administration significantly increased serum TP, GLB, IgY, HDL-C, and T-AOC levels. Serum AST levels were also markedly reduced, and a non-significant trend of decreased A/G (*p* = 0.055) was observed after supplementation with CE (Figure 1A). Furthermore, CE administration could enhance humoral immunity in hens by increasing serum-specific antibody titers against NDV and avian influenza H9 strains (Figure 1B).

### Reproductive Performance

Reproductive performance is a vital indicator in breeding poultry, which affects the economic effectiveness of breeder companies. Descriptive data on the reproductive performance of aged breeder hens are shown in Figure 1C. The rate of fertilization and hatching of fertile (HoF) value was significantly improved upon CE supplementation (*p* < 0.05).

### Intestinal Bacterial Richness, Diversity, and Similarity

**FIGURE 2:**
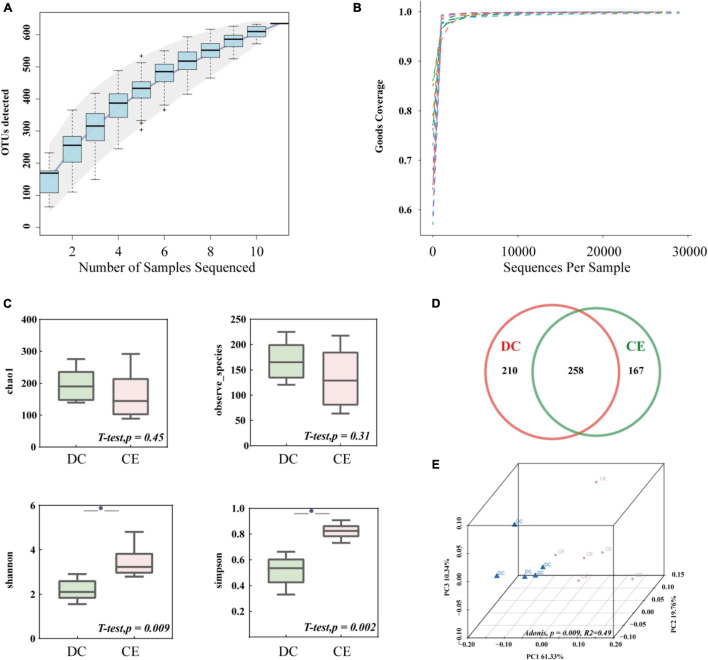
The microbial community structure in the ileum was significantly altered by adding CE (*n* = 6 hens/group). **(A)** Species accumulation curve is used to estimate the rationality of sequencing sample quantity. **(B)** Alpha diversity Rarefaction curve based on Good’s Coverage value, which reflects the rationale of sequencing depth. **(C)** Alpha-diversity evaluation of ileum microbial richness and evenness by measuring chao1, observe-species, Shannon, and Simpson’s diversity indexes. **(D)** Venn diagram is used to represent the amount of shared and unique OTUs numbers. **(E)** Principal coordinate analysis (PCoA) is used to determine the similarities of microbial communities between different groups. *P < 0.05 compared to two groups.

After size filtering, quality control, and chimera removal, an average of 29,200 clean tags and 27,376 valid tags were harvested from each sample for subsequent analysis through 16S amplicon sequencing. The species accumulation curve (Figure 2A) and alpha diversity rarefaction curve (Figure 2B) reached a stable plateau under the sample size and sequencing depth. The alpha diversity index reflects the richness and uniformity of the species composition. The Chao1 and Observed species indices are estimators of phylotype richness, and Shannon and Simpson’s diversity indices reflect both richness and community uniformity. In this study, Shannon and Simpson’s diversity indices were significantly enhanced (*p* < 0.05), while Chao1 and Observed species had a minimal effect on the addition of CE (Figure 2C). The Venn diagram showed that 635 distinct OTUs were clustered based on 97% sequence similarity, among which 258 were shared by both groups (Figure 2D). PCoA based on weighted UniFrac similarity showed a separation of each group (Figure 2E), with 61.33, 19.76, and 10.34% variation explained by principal components: PC1, PC2, and PC3, respectively (Adonis, *p* = 0.009, *R*^2^ = 0.49).

### Ileal Microbial Community Structure

**FIGURE 3:**
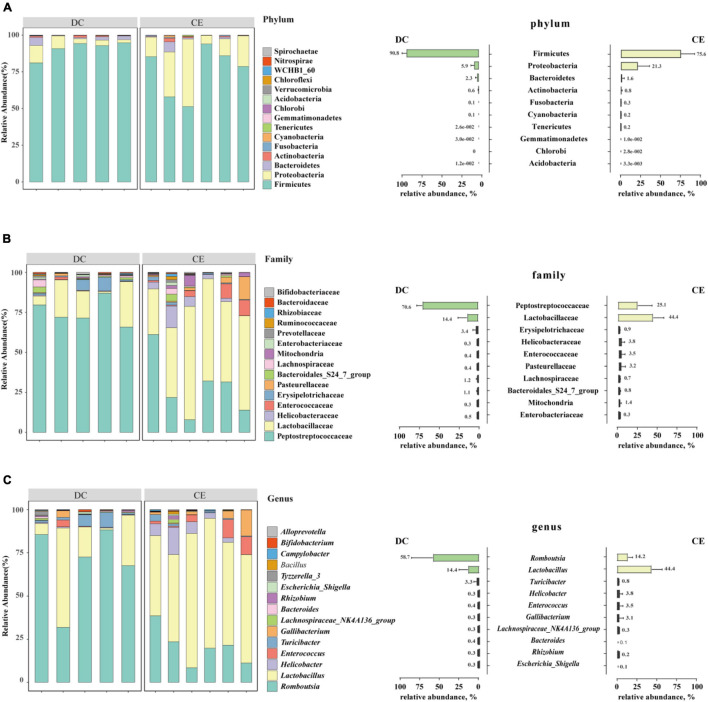
The stacked graph of microbial community structure at **(A)** phylum level, **(B)** family level and **(C)** genus level (*n* = 6 hens/group). The bar chart on the right represents the relative abundance distribution of TOP 10 bacteria at different taxonomic levels, respectively.

Firmicutes, Proteobacteria, and Bacteroidetes were the dominant phyla in the aged breeder hens (relative abundance>1%), accounting for more than 98% of the total bacterial community (Figure 3A). The relative abundance of Proteobacteria increased from 5.94 to 21.05%, and the proportion of Firmicutes decreased from 90.79 to 75.87% with CE supplementation.

At the family level, the phyla of Firmicutes mainly contained Lactobacillaceae, Peptostreptococcaceae, Enterococcaceae, Erysipelotrichaceae, and Lachnospiraceae. Proteobacteria consisted of Helicobacteraceae, Pasteurellaceae, and mitochondria, while Bacteroidetes specifically included the Bacteroidales_S24_7_group (Figure 3B) (relative abundance>1%). Lactobacillaceae and Peptostreptococcaceae were the dominant bacteria in the two groups, and their relative abundances in CE and DC were 44.42 vs. 14.38% and 25.07 vs. 70.57%, respectively.

At the genus level, *Romboutsia, Lactobacillus, Turicibacter, Enterococcus, Gallibacterium*, and *Helicobacter* were the predominant genera in the two groups (Figure 3C) (relative abundance>1%). With the addition of CE, the relative abundance of *Lactobacillus* and *Enterococcus* increased, while the relative abundance of *Romboutsia* decreased.

### Key Microbial Identification

**FIGURE 4:**
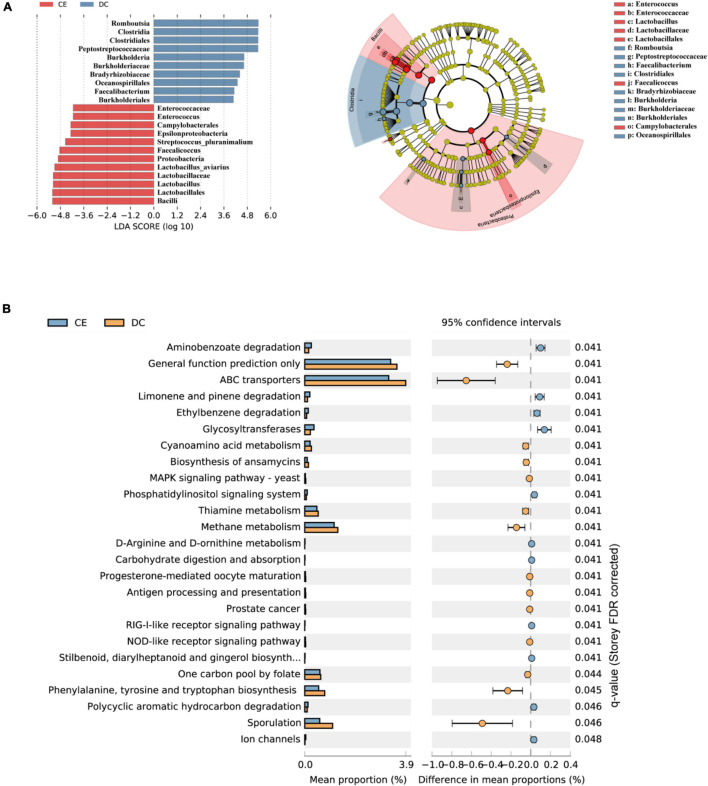
DC and CE have differential bacteria composition and functional preferences (*n* = 6 hens/group). **(A)** LEfSe analysis was performed to identify the bacteria that are differentially represented among the two groups. **(B)** Microbial functional analysis was conducted by PICRUSt software under different experimental conditions.

LDA and effective size comparisons (LEfSe) were conducted to identify the core taxa most likely to explain the differences between the groups. The CE-treated samples appeared to be dominated by *Lactobacillus, Enterococcus, Faecalicoccus*, and *Streptococcus*, whereas DC samples showed *Romboutsia, Faecalibacterium*, and *Burkholderia* as the dominant genera (Figure 4A).

### Predicted Functions of Ileal Bacterial Communities

Significant differences in the gut microbiota were observed between the two groups; however, their functions remain unknown. Hence, we performed a PICRUSt analysis to predict the potential functions of the gut microbiota. All functional genes were divided at level 3. When filtered for non-bacterial functional pathway, the predicted metabolic functional categories in the CE-fed group were related to pathways of biodegradation and metabolism of several xenobiotics, such as “polycyclic aromatic hydrocarbon degradation,” “aminobenzoate degradation,” and “ethylbenzene degradation.” The CE group was also enriched for pathways such as “glycosyltransferases,” “carbohydrate digestion and absorption,” and “D-Arginine, and D-ornithine metabolism.” Pathways such as “sporulation,” “cyanoamino acid metabolism,” “biosynthesis of ansamycins,” “thiamin metabolism,” and “methane metabolism” were enriched in the DC group (Figure 4B).

### Response of Ileum Metabolomic Profiles to Corn Enzyme Diet

**FIGURE 5:**
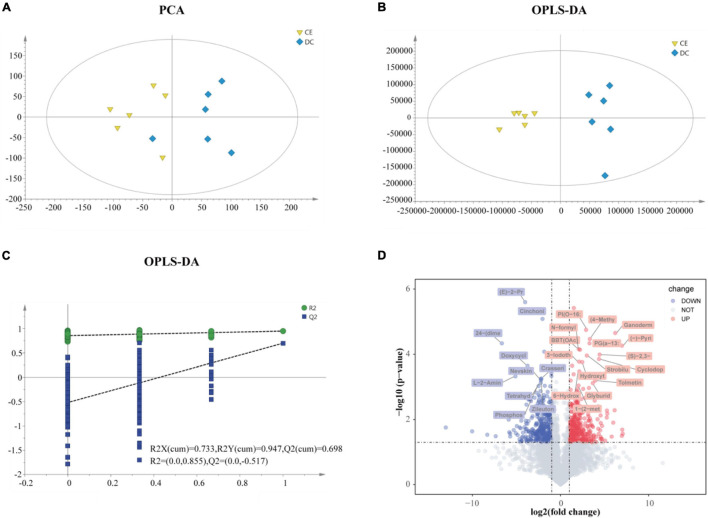
The gut metabolites were remarkably perturbed by adding CE (*n* = 6 hens/group). **(A)** Principal component analyses (PCA) of ileum metabolome. **(B)** Orthogonal projections to latent structures-discriminate analysis (OPLS-DA) score plot was performed on ileum in DC and CE groups. In the permutation validation plot **(C)** (200 cycles) the *Y*-axis intercepts of *R*^2^ and *Q*^2^ are 0.855 and −0.517, respectively, indicating that the model is valid. **(D)** Volcano plots of ileum profiles showing log_2_(fold-change) and −log_1__0_(*p*-value) in metabolites levels induced by adding CE (up-regulated in red and down-regulated in green). The labeled metabolites were of particular interest (| fold change| > 4, *p* < 0.001).

The ileal metabolome was analyzed in both groups to investigate the effect of multi-enzyme supplementation on the ileal chyme. LC-MS detected 23,595 untargeted peaks, and 4,884 metabolites were annotated. To reduce dimensionality, we applied PCA and OPLS-DA to leverage both unsupervised and supervised dimensionality reduction techniques to achieve this goal. Both PCA and OPLS-DA showed separation and discrimination (Figures 5A,B). The quality parameter values of the OPLS-DA model were predicted to be [R2X (cum) = 0.733, R2Y (cum) = 0.947] and fitness [Q2 (cum) = 0.698], which indicated that the model had good reliability and predictability (Figure 5C). The volcano plot indicated up-and downregulated differential metabolites based on statistical values (*p* < 0.05, | log_2_FC| > 1), and *p* < 0.001, | log_2_FC| > 2 was considered to have higher significance (Figure 5D).

### Identification of Differential Metabolites and Critical Metabolic Pathways

**FIGURE 6:**
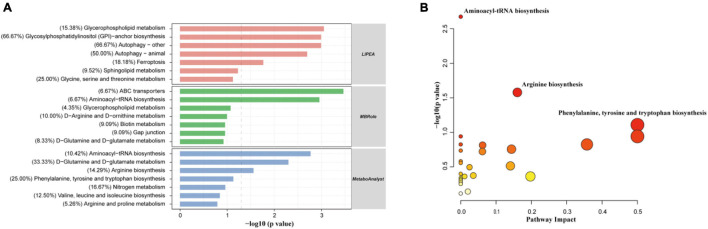
Differential metabolites were significantly enriched in multiple pathways, suggesting that the addition of CE remarkably perturbed gut metabolic activities (*n* = 6 hens/group). **(A)** Functional KEGG pathway enrichment analysis of the differential expressed metabolites was carried out through three different platforms (LIPEA, MBRole, and MetaboAnalyst). **(B)** Analysis of metabolic pathways of DC and CE groups of differentially expressed metabolites was shown by bubble plot, each dot represents a metabolic pathway. *X*-axis and *Y*-axis stand for pathway impact and −log_1__0_(*P*-value), respectively.

In total, 180 differential metabolites were assigned based on VIP values (VIP > 1) and *p*-values (*p* < 0.05). The results of MBRole and MetaboAnalyst (Figure 6A) showed that the differential metabolites were enriched in the “aminoacyl-tRNA biosynthesis,” “ABC transporters,” “D-glutamine and D-glutamate metabolism,” and “arginine biosynthesis pathway.” Moreover, the “arginine biosynthesis pathway” was the most prominent position in the topological analysis (Figure 6B). The LIPEA results (Figure 6A) indicated that the following pathways were significantly enriched by inputting differential lipid metabolites: “glycerophospholipid metabolism,” “glycosylphosphatidylinositol (GPI)-anchor biosynthesis,” “autophagy—other,” “autophagy—animal,” and “ferroptosis” pathways.

### Co-occurrence Patterns of Microbial Communities

**FIGURE 7:**
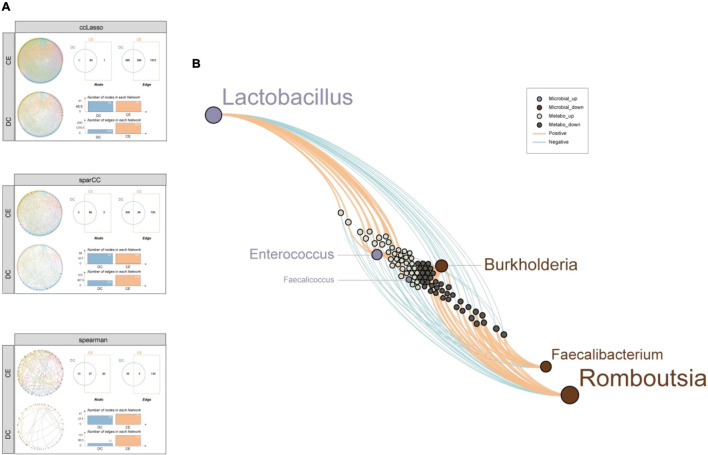
CE enhanced co-expression correlation among the microbial community and the differential metabolites were tightly associated with the signature microbiota. **(A)** Microbial community co-expression network analysis explores the relationship between CE and DC groups through three standard algorithms. Different nodes represent different bacterial genera, colors represent phyla, and the dots size represent node degree. **(B)** A network diagram of differential microbiota-metabolites demonstrates that the up-regulated microbial were closely related with the up-regulated metabolites while down-regulated microbial were closely related with the down-regulated metabolites.

To further explore the complex microbial community structures in the DC and CE groups, we performed co-occurrence network analysis by calculating CCLasso (Fang et al., 2015), sparCC (Friedman and Alm, 2012), and NAMAP with Spearman correlation inference algorithm *via* MetagenoNets between microbial taxa at the genus level based on 16S sequencing (Nagpal et al., 2020; Figure 7A). The results showed that the addition of CE significantly increased the interrelationship between bacteria under all three algorithms [edges: 2,541 vs. 954; 815 vs. 425; 137 vs. 33, CE vs. DC (CCLasson, SparCC, Spearman, respectively)], while the number of correlated nodes did not change significantly [nodes: 91 vs. 90; 88 vs. 89; 47 vs. 39, CE vs. DC (CCLasson, SparCC, Spearman, respectively)]. CCLasso obtained the highest number of interrelationships, followed by the SparCC and Spearman algorithms. All three algorithms indicated that CE activated the interactions between bacteria. Different algorithms have unique advantages and shortcomings. SparCC (Friedman and Alm, 2012) is a microbial network algorithm developed based on the log-ratio transformation of compositional data, which solves the problem of poor performance of the Spearman algorithm under the sparsity condition of bacterial communities; however, it did not consider the influence of errors in the compositional data (Fang et al., 2015). CCLasso made improvements based on such issues and had the characteristic of better edge recovery.

### Correlations Among Differential Microbiota and Metabolites

Constructing a network between differential microbiota and metabolites is important for understanding their interaction relationships. Spearman correlation analysis of six microbiota (by LEfSe, LDA > 4, *p* < 0.05) and 180 metabolites (by *p* < 0.05, VIP > 1) was conducted (Figure 7B). The results showed that the bacteria enriched in CE were remarkably correlated with the upregulated metabolites. In contrast, the bacteria enriched in DCs were remarkably correlated with downregulated metabolites, reflecting a clear differential interaction pattern. This result further demonstrated a significant change in microbe-mediated metabolic patterns after the addition of CE.

**FIGURE 8:**
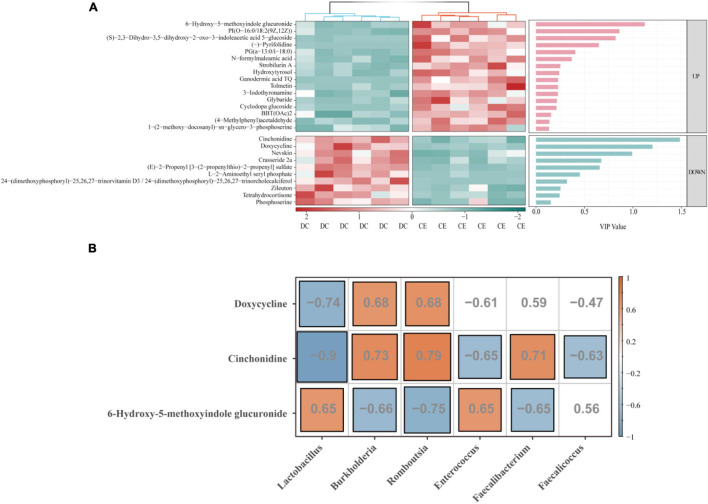
Correlation analysis was conducted between the top focus metabolites and the signature microbiota to explore the key factors influencing organismal fitness by adding CE. **(A)** Heat map of differential metabolites with a bar graph of VIP values from previous OPLS-DA model. Only if the metabolites were a satisfied condition of VIP > 1 were selected for the following association analysis. **(B)** The correlation of top focus metabolites and the signature microbiota.

To further identify more specific and sensitive markers of metabolites, we performed a more stringent threshold criteria (*p* < 0.001, | FC| > 4, VIP > 1) (Figure 8A). The top focus metabolites were used to perform correlation analysis with the signature microbiota (LDA > 4, *p* < 0.05) (Figure 8B). The results showed that *Lactobacillus* was significantly positively correlated with 6-hydroxy-5-methoxyindole glucuronide and negatively correlated with doxycycline and cinchonidine, while *Romboutsia* and *Burkholderia* had the opposite regulation pattern to *Lactobacillus*. In addition, both *Enterococcus* and *Faecalicoccus* were negatively correlated with cinchonidine, and *Enterococcus* was also significantly positively correlated with 6-hydroxy-5-methoxyindole glucuronide.

## Discussion

Enzyme supplementation of poultry feed is of great significance in nutrition, economics, and the environment. Enzymes can improve the utilization of carbohydrates, proteins, lipids, and phytate phosphorus in feed to reduce the waste of fodder values and pollutant emissions (Douglas et al., 2000; Dosković et al., 2013). Our study showed that supplementation with multiple enzymes had no significant effect on laying performance, which contrasted with the results of previous studies. Studies by Khan et al. (2011) showed that adding 2.0 g/kg multi-enzyme preparation can increase egg production, egg weight, and egg mass; and improve the feed conversion ratio and bodyweight of layers without changing feed intake. Although enzymes have a significant impact on the performance of poultry, their application is greatly limited by the wide variety of enzymes and harsh application conditions. A previous study showed that the addition of complex enzymes (phytase, xylanase, cellulase, α-amylase, and acid-protease) had little effect on the production performance of aged hens (60–68 weeks old) but increased intestinal enzyme activity and nutrient retention (Wen et al., 2012). Interestingly, adding enzymes to low-protein and low-AME diets significantly improves hen and broiler performance and digestive enzyme activities (Zhou et al., 2009; Zhu et al., 2014; Rehman et al., 2018). Therefore, we supposed that adding multi-enzyme preparations has little impact on production performance, partly due to the balanced nutrition diet and the health status of breeding hens.

Blood biochemical indices reflect the health status of hens. CE administration increased the levels of TP and GLB in the serum. This was probably due to the adequate degradation of proteins promoted by the enzymes, which improve the absorption and utilization of amino acids in the small intestine (Al-Homidan et al., 2020). IgY is the primary serum antibody mainly distributed in poultry serum and egg yolk to protect the hens and their offspring from pathogens (Thirumalai et al., 2019). The improvement of in the titers of IgY and serum NDV and avian influenza H9 strain antibodies was associated with enhanced disease resistance. Enhanced humoral immunity was possibly related to the immune-regulatory effects of some oligosaccharides and beneficial microbiota in the gut after feeding with multiple enzymes. Additionally, CE administration significantly increased the serum HDL-C and T-AOC content and decreased the AST content. The T-AOC reflects the cumulative effect of all antioxidants in the blood and body fluids (Suresh et al., 2009; Liu et al., 2021). Breeding hens frequently face oxidative stress and ovarian aging problems in the later laying stage, which considerably affect their performance and physiology (Liu et al., 2018). AST is a sensitive marker for detecting liver injury, and high levels of AST indicate liver damage (Yousefi et al., 2005). HDL-C is considered “good cholesterol” and is associated with cardiovascular health. It can accelerate lipid migration from peripheral tissues to the liver, where cholesterol can be metabolized into bile acids (Li et al., 2018; Duan et al., 2019). Therefore, adding multiple enzymes to the diet enhances host systemic immunity, improves antioxidant capacity, and has no adverse effects on liver function.

Egg quality and reproductive performance, two important economic traits for breeding hens, tend to decrease rapidly because of the lower efficiency of absorption and immunity with age (Liu et al., 2001; Bain et al., 2016). We found that adding multiple enzymes can significantly increase albumen height and Haugh unit, indicating that the addition of multiple enzymes improves egg freshness. We then analyzed reproductive performance and found that CE administration also significantly enhanced the rate of fertilization and HoF value. The improvement of these two reproduction indexes may be related to the high-quality protein of the eggs and increased deposition of IgY in the yolk, thereby improving the reproductive performance of aged breeding hens (Thirumalai et al., 2019). In summary, CE supplementation can effectively enhance egg quality and reproductive performance of breeding hens.

To further discern the underlying mechanism of the enzyme on the productive performance and immune function of hens. The gut microbiota and metabolome after enzyme treatment were analyzed. Our results showed that adding CE had minimal effect on Chao1 and Observed species, but significantly increased Shannon and Simpson’s diversity indices. Meanwhile, the PCoA showed a clear separation between the CE and DC groups, which indicated that multiple enzymes could dramatically alter the gut microbiota with increasing microbial evenness without decreasing microbial richness (Zhang et al., 2017). Consistent with the results of previous studies, Firmicutes, Proteobacteria, and Bacteroidetes were the dominant phyla in the ileum of hens, accounting for more than 98% of the total bacteria (Pan and Yu, 2014; Liu et al., 2021). The genera *Lactobacillus*, *Enterococcus*, *Streptococcus*, and *Faecalicoccus* were the signature taxa of the CE group determined using LEfSe (LDA > 4, *p* < 0.05). *Lactobacillus* spp. contribute to intestinal health, immunity enhancement, nutrient absorption, and bile acid hydrolysis (Staley et al., 2017; Xiao et al., 2017). *Enterococcus* spp. are lactic acid bacteria that produce bacteriocins against pathogenic bacteria and regulate nutrient metabolism (Hanchi et al., 2018). *Streptococcus* spp. such as *S. thermophiles* and *S. salivarius* are often considered to have probiotic effects, which help establish intestinal immune homeostasis and regulate the inflammatory response of the host (Akpinar et al., 2011; Kaci et al., 2014). Analysis of microbial co-occurrence network patterns suggested that the addition of multi-enzymes remarkably increased the interactions between gut microbiota without affecting the number of interacting bacteria, illustrating that adding multiple enzymes enhanced the communication between bacteria. Correlation analysis of differential microbiota and metabolites demonstrated that the gut microbiota signature genera were strongly correlated with altered metabolites. Therefore, the addition of multi-enzyme modulated immune function and metabolism may be related to altering the intestinal microbiota, increasing the relative abundance of potentially beneficial bacteria, and enhancing the interaction between bacteria.

The gene function analysis of the predicted metagenomes from the DC group suggested that the microbial pathways were significantly enriched in the sporulation and biosynthesis of ansamycins. Spores can store the microbiota’s hereditary material in a harmful or unsuitable environment so that their metabolism in this state is 10 million times slower than in normally growing bacteria (Huang and Hull, 2017; Bressuire-Isoard et al., 2018). Ansamycins are antibiotics produced by several *Actinomycetes* strains and have an inhibitory effect on the growth of many bacteria (Vardanyan and Hruby, 2016). Bacteria inhibit the growth of their surrounding bacteria by synthesizing antibiotics to compete for limited resources, leading to a vicious cycle in the gut environment. Metabolic pathway enrichment analysis showed significant enrichment of several pathways, including glycerophospholipid metabolism, autophagy, and ferroptosis. This could be because the bacteria in the DC group lacked genes related to the degradation of harmful substances and the higher concentration of antibiotics surrounding them. Hence, bacteria may degrade their components or excess proteins through autophagy to provide nutrition for survival or directly induce ferroptosis-like death in the DC group (Deretic and Levine, 2009; Xu et al., 2019; Shen et al., 2020). Spearman correlation analysis revealed that two top-focused metabolites, doxycycline, and cinchonidine, enriched in the DC group, were positively correlated with the DC signature bacteria *Romboutsia* spp. and *Burkholderia* spp. Doxycycline, a tetracycline, has a bacteriostatic effect by inhibiting the synthesis of bacterial proteins by destroying transfer RNA and messenger RNA at ribosomal sites (Raval et al., 2018). Because doxycycline is significant for maintaining animal health and controlling vertically transmitted diseases, it has been widely used in the breeding industry (Yan et al., 2018). Studies have shown that doxycycline mainly affects the relative abundance of Firmicutes and Proteobacteria and reduces the richness and evenness of the flora (Boynton et al., 2017; Stavroulaki et al., 2021). Cinchonidine is an alkaloid found in several foods such as fruits, herbs, spices, and olives (Olea europaea) (Eyal, 2018). However, the biosynthetic pathway of cinchonidine remains unclear (Maldonado et al., 2017). Overall, the bacteria in the DC group enriched genes related to sporulation and biosynthesis of ansamycins pathways and lacked communication. The intestinal environment had a higher doxycycline content than the CE group, which would affect the microbial community structure and reduce the evenness (Stavroulaki et al., 2021).

The gene function analysis of the predicted metagenomes from the CE group suggested that the microbial pathways were significantly enriched in the biodegradation and metabolism of multiple harmful substances. Polycyclic aromatic hydrocarbons (PAHs) are widely distributed organic pollutants with genetic toxicity and carcinogenicity that can significantly interfere with gut microbiota and are associated with harmful effects on host health (Ghosal et al., 2016; Redfern et al., 2021). Ethylbenzene is a toxic aromatic organic compound metabolized by the organism, and the accumulation of xenobiotics in an organism may cause tissue damage and harm the host (Pan et al., 2020). The addition of multiple enzymes significantly enriched microbial functional genes related to the degradation of the aforementioned harmful substances, which suggested that the microbes of the CE group may have a better ability to degrade toxic organic compounds and maintain homeostasis of the gut environment to create a better intestinal environment. Meanwhile, the ileum microbiota in the CE group also enriched “glycosyltransferases” pathways, which may promote bacterial surface antigen formation, thus stimulating the host immune system and improving humoral immunity (Hong et al., 2019). Spearman correlation analysis revealed that one top-focused metabolite, 6-hydroxy-5-methoxyindole glucuronide, enriched in the CE group, was positively correlated with the CE signature bacteria *Lactobacillus* spp., and *Enterococcus* spp. 6-Hydroxy-5-methoxyindole glucuronide, a member of the glucuronide family. It is a natural metabolite of 6-hydroxy-5-methoxyindole generated in the liver by UDP glucuronyltransferase, which assists with the excretion of toxic substances, drugs, or other substances that cannot be used as an energy source (Zhao et al., 2012; Liu et al., 2019). Collectively, the addition of multiple enzymes can improve the ability of microbes to degrade harmful substances, and the potentially beneficial bacteria enriched in the CE group are closely related to the metabolite 6-Hydroxy-5-methoxyindole glucuronide that facilitates the excretion of toxic substances. Thus, CE addition can benefit hen health, possibly by affecting the metabolic function of intestinal bacteria.

Taken together, the results showed that CE supplementation may provide a nutrient-rich environment for bacteria by improving the digestion and absorption of starch and protein, elevating the excretion of toxins and harmful substances, and reshaping the structure of the ileal microbial community such that *Lactobacillus* spp. are the dominant bacteria and the relative abundance of common potentially beneficial bacteria, such as *Enterococcus* and *Streptococcus*, is increased. Follow-up studies are needed to ascertain the changes in the gut microbiome and metabolome induced by complex enzymes on intestinal cell function.

## Conclusion

Overall, administration of 0.2 g/kg of dietary multi-enzyme could enhance humoral immunity and improve egg quality and reproductive efficiency together with intestinal microbial community structure and metabolite composition of aged breeding hens. Multi-enzymes could be used to enhance the immunity and reproductive performance of old breeding hens and extend their service life.

## Data Availability Statement

The microbial raw sequencing data and the metabolome data were deposited into the NCBI Sequence Read Archive database (SRA accession: PRJNA728385).

## Ethics Statement

The animal study was reviewed and approved by Animal care and Use Committee of China Agricultural University.

## Author Contributions

ZW and ZN conceptualized the project. YL performed experiments, collected data, analyzed data, and wrote the manuscript. LQ designed studies and made insightful edits. DZ performed experiments. All authors reviewed the manuscript and approved the final submission.

## Conflict of Interest

DZ was employed by company Huayu Agricultural Science and Technology Co., Ltd. The remaining authors declare that the research was conducted in the absence of any commercial or financial relationships that could be construed as a potential conflict of interest.

## Publisher’s Note

All claims expressed in this article are solely those of the authors and do not necessarily represent those of their affiliated organizations, or those of the publisher, the editors and the reviewers. Any product that may be evaluated in this article, or claim that may be made by its manufacturer, is not guaranteed or endorsed by the publisher.

## Footnotes

1
http://huttenhower.sph.harvard.edu/galaxy/

2
http://picrust.github.io/picrust/

3
https://www.genome.jp/kegg/ko.html

4
https://hmdb.ca/

5
http://www.lipidmaps.org

6
http://metlin.scripps.edu

7
https://www.metaboanalyst.ca/

8
http://csbg.cnb.csic.es/mbrole2/

9
https://lipea.biotec.tu-dresden.de/home
